# Environmental Concentrations of PFOS Accumulate in the *Euglena* Eyespot and Impair Chloroplast ATP Synthase Activity: A Dual Impairment of Phototaxis and Photosynthetic Light Reactions

**DOI:** 10.3390/toxics14060540

**Published:** 2026-06-22

**Authors:** Peirui Liu, Junfeng Wang, Yan Hong, Zilin Chen, Xiaoya Liu, Huayi Chen, Ganning Zeng, Xiangliang Pan

**Affiliations:** 1 Zhejiang Key Laboratory of Low-Carbon Control Technology for Industrial Pollution, College of Environment, Zhejiang University of Technology, Hangzhou 310014, China; 2 School of Environmental Science and Engineering, Xiamen University of Technology, Xiamen 361024, China; 3 School of Tropical Agriculture and Forestry, Hainan University, Haikou 570228, China

**Keywords:** *Euglena gracilis*, PFOS, eyespot, phototaxis, ATP synthase, photophosphorylation, dual impairment

## Abstract

Perfluorooctane sulfonate (PFOS) is a persistent organic pollutant widely detected in aquatic ecosystems, but its subcellular targets and the mechanisms by which it disrupts light resource utilization in photosynthetic protozoa remain poorly understood at concentrations spanning environmentally typical to supra-environmental levels. Here, *Euglena gracilis* G.A. Klebs was exposed to PFOS at concentrations spanning environmentally typical (0.5 µg/L), hotspot-relevant (5 µg/L), and supra-environmental (50 µg/L) levels. Subcellular distribution, phototaxis, photosynthetic light reactions, and energy metabolism were investigated using isolated chloroplast assays, transcriptomics, and proteomics. TEM-EDS mapping revealed pronounced fluorine signal enrichment, attributable to PFOS, in the eyespot and chloroplasts. Eyespot fluorine enrichment was associated with impaired phototactic motility and an altered light perception threshold. PFOS did not acutely inhibit the maximum photochemical efficiency of photosystem II (*Fv*/*Fm*); instead, a transient upregulation of photosynthesis-related genes was observed, which weakened with prolonged exposure, whereas the photosynthetic electron transport rate (ETR) was significantly reduced. PFOS significantly reduced ATP levels and *ETR*, while *Fv*/*Fm* remained unchanged and non-photochemical quenching (*NPQ*) was elevated. Isolated chloroplast assays revealed that PFOS inhibits Mg^2+^-dependent ATP hydrolytic activity in the chloroplast-enriched fraction and impairs thylakoid electron transport, consistent with impaired chloroplast ATP synthase function, though the specific molecular target and mechanism remain to be conclusively demonstrated. Transcriptomic and proteomic analyses revealed compensatory upregulation of photosynthesis pathways but suppression of ATP synthesis and redox homeostasis. Collectively, our results suggest that PFOS impairs chloroplast ATP synthase function, accompanied by reduced ETR and elevated NPQ. Together with the eyespot-associated phototaxis impairment, these effects suggest that PFOS may dually disrupt light acquisition (behavioral) and light conversion (physiological) in *E. gracilis*. This dual impairment may compromise the ecological fitness of *Euglena* in PFOS-contaminated environments, especially under prolonged exposure. It should be noted that the subcellular fluorine mapping is qualitative, the phototaxis assay reflects population-level responses, and the ATP synthase impairment interpretation is indirect; the proposed mechanistic model remains a hypothesis requiring further direct experimental validation.

## 1. Introduction

Perfluorooctane sulfonate (PFOS), a representative persistent organic pollutant of per- and polyfluoroalkyl substances (PFAS), is widely detected in surface waters at concentrations typically below 300 ng/L, but can exceed this level in contaminated hotspots [1,2,3,4,5,6,7]. PFOS has been extensively used in industrial applications including firefighting foams, metal electroplating, and water-repellent coatings, and enters natural water bodies primarily through industrial wastewater discharge, fire-training site runoff, and wastewater treatment plant effluents, representing a major source of perfluorinated compound pollution in aquatic ecosystems [8]. Owing to its chemical stability and environmental persistence, PFOS has been listed under the Stockholm Convention on Persistent Organic Pollutants for global regulation [9]; nevertheless, elevated concentrations continue to be detected in certain natural water bodies, sustaining ongoing ecological risks and underscoring the practical necessity of toxicity investigations [10]. Unlike classical lipophilic pollutants, PFOS exhibits surfactant properties and a strong affinity for proteins, preferentially binding to fatty acid-binding proteins and albumin [11]. This protein-binding behavior suggests that PFOS may accumulate in protein-rich subcellular structures.

*Euglena gracilis* was selected as the model organism because it uniquely possesses an eyespot, flagella, and chloroplasts within a single cell, enabling simultaneous assessment of multiple toxicity endpoints including photosynthetic activity, phototactic response, and motility. It is a widely used unicellular model organism in environmental toxicology and photobiology research [12]. At the anterior end of the cell, *Euglena* possesses a stigma (eyespot) composed of a lipoprotein complex rich in carotenoids and rhodopsin-like proteins, which sense light intensity and direction and modulate flagellar movement to drive phototaxis [13,14]. The photoactivated adenylyl cyclase (PAC) within the eyespot is a core phototransduction protein; upon blue-light stimulation, PAC promotes cAMP production, thereby regulating flagellar motility [13]. Phototactic behavior determines the spatial positioning of cells in a light environment and thus influences the efficiency of light resource acquisition in *Euglena* [12].

Preliminary observations using transmission electron microscopy combined with energy-dispersive X-ray spectroscopy mapping (TEM-EDS mapping) revealed that after PFOS exposure, fluorine signal intensity was notably enhanced in the eyespot region, suggesting local enrichment. Most existing studies on the effects of PFOS on photosynthetic organisms have focused on direct chloroplast damage under relatively high concentrations [15,16], whereas little attention has been paid to whether the eyespot, a photoreceptive structure, is also affected. Moreover, direct molecular evidence for an interaction between PFOS and the eyespot photoreceptor PAC remains lacking. Given the eyespot’s critical role in light perception and energy acquisition, understanding whether PFOS targets this organelle at environmentally relevant concentrations is essential for assessing its ecological risks to photosynthetic flagellates.

The present study therefore addresses the following questions: (i) Does environmentally relevant PFOS accumulate in the *Euglena* eyespot and concurrently induce phototactic abnormalities? (ii) Does PFOS directly affect chloroplast energy conversion? (iii) What is the relationship between eyespot-mediated behavioral impairment and photosynthetic physiological changes? To address these questions, we examined the subcellular distribution of PFOS using TEM-EDS mapping, analyzed its potential interaction with PAC via molecular docking, and assessed eyespot function through phototactic motility, flagellar shedding rate, and apparent migration velocity. Photosynthetic and metabolic status were evaluated by measuring chlorophyll fluorescence parameters (including *Fv*/*Fm*, *ETR*, *NPQ*), chlorophyll content, intracellular ATP and ROS levels. Isolated chloroplast assays were performed to distinguish direct from indirect effects, and transcriptomic and proteomic analyses were used to characterize molecular response signatures.

## 2. Materials and Methods

### 2.1. Cultivation of Euglena gracilis and PFOS Exposure

*Euglena gracilis* (*E. gracilis*) strain FACHB-848 was obtained from the Institute of Hydrobiology, Chinese Academy of Sciences. Stock cultures were maintained in sterile EM medium under controlled conditions: a 12 h:12 h light–dark cycle with 500 K cool-white LED illumination (MBTL-T8-18, rated power 18 W; photosynthetic photon flux density (PPFD) of approximately 52.5 µmol·m^−2^·s^−1^, corresponding to 3500 lux; the blue-light component has a dominant wavelength of approximately 450–460 nm with a half-peak bandwidth of ~20–30 nm)at a temperature of 25 ± 1 °C. Cultures were incubated under the aforementioned light and temperature regime until they reached the logarithmic growth phase, and the initial optical density at 680 nm (OD_680_) of the culture was adjusted to 0.1 using a UV–visible spectrophotometer (UV1800, Shimadzu Instruments Co., Ltd., Kyoto, Japan). OD_680_ was selected because chlorophyll a lipoprotein particles in *Euglena* chloroplasts exhibit a characteristic absorption peak at 680 nm [17], and OD_680_ has been validated to correlate well with cell density in green microalgae for toxicological growth monitoring [18]. The relationship between OD_680_ and cell density was determined by a standard curve: cell density (×10^4^ cells/mL) = 65.042 × OD_680_ + 1.5846 (R^2^ = 0.9996). Subsequently, a stock solution of PFOS was added to achieve final exposure concentrations of 0.0, 0.5, 5.0, and 50.0 μg/L. Based on field monitoring data [5,7] and reported algal toxicity threshold concentrations, 0.5 µg/L represents a mildly contaminated ambient water concentration (environmentally typical), 5 µg/L represents the reported threshold for initiating physiological responses in algae (hotspot-relevant threshold), and 50 µg/L was selected to simulate industrial discharge or extreme accumulation scenarios (supra-environmental/hotspot-extreme). All subsequent experiments were performed using cells derived from at least three independent culture batches for each trophic condition to ensure biological reproducibility.

#### 2.1.1. PFOS Quantification

PFOS concentrations were quantified using ultra-performance liquid chromatography (UPLC, ACQUITY UPLC I-Class, Milford, MA, USA) coupled with tandem mass spectrometry (XEVO TQ-S, Waters, Milford, MA, USA). The system was equipped with an ACQUITY UPLC BEH C18 column (2.1 mm × 100 mm, particle size 1.7 μm, Waters, Milford, MA, USA) and an electrospray ionization (ESI) source. The mobile phase consisted of 5 mmol/L ammonium acetate (solution A) and 100% methanol (solution B). Samples were directly injected into the UPLC system and eluted at a flow rate of 0.2 mL/min. Mass spectrometry was performed in negative ESI mode with optimized conditions as follows: drying gas (N_2_) temperature 300 °C, drying gas flow 6 L/min, sheath gas (N_2_) temperature 260 °C, sheath gas flow 11 L/min [19]. LC-MS data were acquired in multiple reaction monitoring (MRM) mode and processed using MassLynx 4.1 software. A standard curve was constructed using PFOS standard solutions at concentrations of 1.0, 5.0, 10.0, 50.0, and 200.0 μg/L, yielding an R^2^ of 0.9995. Prior to use, PFOS was further calibrated by gas chromatography–tandem mass spectrometry (LC-MS/MS). All glassware used in the experiment was sequentially washed with methanol and ultrapure water, then ultrasonicated, dried, wrapped in aluminum foil, and baked in a muffle furnace at 350 °C for 4 h.

#### 2.1.2. Visualization of PFOS Accumulation in the Eyespot

*E. gracilis* cells were fixed in 2.5% (*v*/*v*) glutaraldehyde for two days. The cells were then suspended in 2.5% warm agar. After solidification, small cubes were cut from the sample and post-fixed in 1% (*w*/*v*) OsO_4_ for 1 h. The fixatives were buffered with 0.07 M Na_2_HPO_4_-KH_2_PO_4_ (pH 7.2). Following dehydration in a graded ethanol series, the samples were embedded in Durcupan ACM resin. Ultrathin sections were cut using a Reichert Jung Ultracut M microtome (Reichert-Jung Ltd., Vienna, Austria), mounted on copper grids, stained with 5% uranyl acetate and Reynold’s lead citrate solution, and observed under a transmission electron microscope (TEM) [20]. The culture medium, reagents, embedding resin, and all sample preparation procedures contained no fluorine-containing components; the sole exogenous fluorine input was the experimentally added PFOS. Therefore, fluorine signals detected in cellular structures can be specifically attributed to PFOS. TEM-EDS mapping in this study was employed for qualitative characterization of fluorine spatial distribution and relative enrichment patterns.

#### 2.1.3. AFM-IR Analysis of C–F Bond Signals in PFOS-Exposed Cells

To confirm the presence of C–F bonds (characteristic of PFOS) in exposed cells and to provide orthogonal supporting evidence for the TEM-EDS fluorine mapping results, atomic force microscopy–infrared spectroscopy (AFM-IR) analysis was performed using an Anasys nanoIR2 system (Bruker Technologies, Billerica, MA, USA). The C–F bond infrared absorption peak of PFOS was first identified by comparing the FT-IR spectra of PFOS and polytetrafluoroethylene (PTFE), confirming the C–F stretching vibration at approximately 1247 cm^−1^ (Figure S1a). For AFM-IR point spectroscopy, cells from control and PFOS-exposed (50 µg/L, 7 days) groups were deposited on clean silicon substrates and air-dried. Point spectra were collected at multiple positions on the cell surface in the range of 1000–1800 cm^−1^ to identify the C–F absorption signal (1248–1252 cm^−1^), the amide II band of proteins (1640–1644 cm^−1^), and the ester carbonyl band of lipids (1728–1732 cm^−1^) (Figure S1b). Nano-IR mapping was subsequently performed at these characteristic wavenumbers with a spatial resolution of approximately 20 nm. Image analysis was conducted using Analysis Studio V 3.15 software. C–F bond signal intensity was compared between control and PFOS-exposed cells. Three independent biological replicates were analyzed, with at least five cells scanned per replicate. It should be noted that AFM-IR detects the C–F bond vibrational signal rather than intact PFOS molecules, and the signal intensity analysis is semi-quantitative. Therefore, AFM-IR results are used as supporting evidence complementing the TEM-EDS mapping data.

#### 2.1.4. Molecular Docking Analysis

To predict the binding mode between PFOS and the photoreceptor protein photoactivated adenylyl cyclase (PAC) in the *Euglena* eyespot, the amino acid sequence of PAC was retrieved from the UniProt database. As no crystal structure of PAC is currently available, a dimeric model was generated using ProteniX base v1.0.0 (Bytedance, Beijing, China) and subsequently energy-minimized with Rosetta Relax. The obtained three-dimensional (3D) protein structure was preprocessed using PyMOL 2.5.4 (Schrödinger, Inc., New York, NY, USA) software by removing water molecules and adding hydrogen atoms, and the final structure was saved in PDB format. The SMILES string of PFOS was obtained from the PubChem database, and its 3D structure was optimized using Python 3.10 with the RDKit toolkit based on the MMFF94 force field. Molecular docking was performed using CB-DOCK2 software with AutoDock Vina 1.2.3 (The Scripps Research Institute, La Jolla, CA, USA) for blind docking [21]. CB-DOCK2 employs an artificial neural network for cavity detection and uses AutoDock Vina to perform the docking calculations. The optimal protein–ligand complex was selected based on the predicted binding energy (calculated with the AMBER force field), followed by analysis of intermolecular interactions and evaluation of the docking pose. Non-covalent interactions between the ligand and receptor were analyzed using PLIP 2.3.0 [22], and the 3D conformational diagrams were visualized with PyMOL (version 2.5.4). It must be emphasized that, in the absence of an experimentally resolved PAC structure and without functional validation through measurement of light-induced cAMP production or PAC-dependent signaling assays, these molecular docking results are purely theoretical predictions and can only serve to generate scientific hypotheses; they are insufficient to confirm that PFOS binds PAC in vivo or inhibits PAC function. Functional validation through measurement of light-induced cAMP production, PAC-dependent signaling, and phototactic responses under defined blue-light stimulation will be the focus of our subsequent investigations.

### 2.2. Phototactic Behavior Assays

#### 2.2.1. Determination of Apparent Vertical and Horizontal Migration Velocity

**Figure 1:**
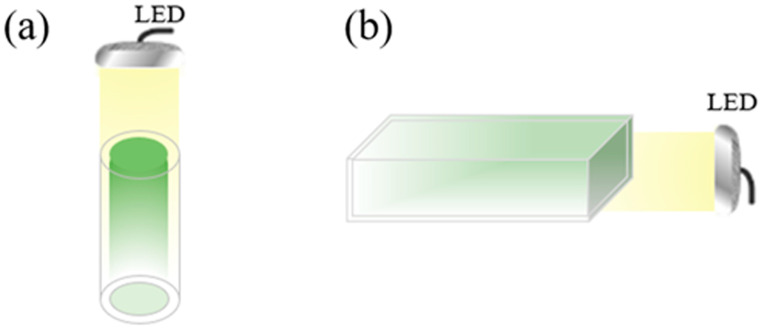
The schematic diagram of the swimming device. (**a**) Vertical movement; (**b**) horizontal movement. Yellow indicates the light source, and green indicates the algal suspension.

A laboratory microscale device was used to measure the apparent migration ability of *E. gracilis* (Figure 1). For vertical movement experiments, a cylindrical acrylic channel with an inner diameter of 4.4 cm and a height of 10.0 cm was employed. The channel was illuminated from above by a 6500 K cool-white LED lamp (PPFD approximately 103.83 µmol·m^−2^·s^−1^) for 20 min, and sampling points were set every 3.0 cm [23]. For horizontal movement experiments, a rectangular acrylic channel with a square cross-section (inner width 4.0 cm, length 9.0 cm) was used. Illumination was provided from the side by a 6500 K cool-white LED lamp (PPFD approximately 185.78 µmol·m^−2^·s^−1^) for 20 min, with sampling points every 3.0 cm. Only the side facing the light source was transparent. The light source was a planar LED panel constrained by light-shielding to produce approximately parallel directional light, establishing a continuous light gradient from the light-proximal to the light-distal end. Light conditions were controlled by fixing the distance between the light source and the chamber and using light-shielding hoods to constrain the beam path. The apparent migration velocity was calculated using the following formula [23]:

$$Velocity=(C_{1}V_{1}\times S_{1}+C_{2}V_{2}\times S_{2}+\ldots \ldots +C_{x}V_{x}\times S_{x})/t/(C_{1}V_{1}+C_{2}V_{2}+\ldots \ldots +C_{x}V_{x})$$

where

*Velocity* = mean apparent phototactic migration velocity of the algal population (µm·s^−1^);

*C* = cell concentration at each sampling position (cells·mL^−1^);

*V* = sampling volume at each position (mL, kept consistent across all segments);

*S* = distance from the sampling position to the initial cell inoculation site (cm, equally spaced at 3 cm intervals along the light gradient direction);

*t* = total duration of the light-induced migration experiment (s, i.e., 1200 s).

Samples were collected by slow, fixed-point pipetting from top to bottom (vertical channel) or from light-proximal to light-distal end (horizontal channel), with a controlled single-sampling volume of 200 µL to minimize aspiration artifacts, convective mixing, and mechanical disturbance. Each sampling position was measured with three biological replicates, with five repeated samplings per replicate (15 measurements per position). The multiple repeated measurements effectively mitigated any perturbation from the sampling process itself, preserving the original cell spatial distribution gradient. The spatial light intensity distribution along the channel was measured using a solar power meter (Model SM206-SOLAR, Xinbao Keyi, Shenzhen, China) at each position (1 cm intervals) under identical conditions (same 6500 K cool-white LED lamp, same chamber geometry, without cell suspension). Irradiance (W·m^−2^) was recorded in triplicate. The measured irradiance values were converted to estimated PPFD (μmol·m^−2^·s^−1^) using an empirical conversion factor predetermined for the 6500 K cool-white LED light source. As shown in Figure S2, the estimated PPFD decreased monotonically from the light-proximal end to the far end. The light spectrum was not independently re-characterized beyond the manufacturer’s specifications, which is a minor limitation; nonetheless, the measured gradient provides a robust quantitative basis for interpreting the phototactic response curves.

#### 2.2.2. Phototactic Movement Assay

For phototaxis experiments, the rectangular acrylic channel described above (Figure 1a) was used. *E. gracilis* cells exposed to PFOS under autotrophic conditions were placed at the end of the channel near the light source. The channel was illuminated from the side by a 6500 K cool-white LED lamp for 20.0 min, with light intensity increasing stepwise from a PPFD of approximately 30 µmol·m^−2^·s^−1^ to approximately 150 µmol·m^−2^·s^−1^ (corresponding to 2000 lux to 10,000 lux) in increments of approximately 30 µmol·m^−2^·s^−1^ (2000 lux). Subsequently, samples were collected from both the near-light end and the far-light end, and the ratio of cell density at the near-light end to that at the far-light end was calculated. Local cell densities were quantified by direct microscopic counting using a hemocytometer, not by OD_680_. The OD_680_–cell density standard curve was used exclusively for initial bulk culture concentration calibration. It should be noted that this endpoint ratio method cannot completely exclude potential interference from reduced cell motility, aggregation, or sedimentation; however, supporting evidence from eyespot TEM-EDS enrichment and the gradient-dependent nature of the phototactic response indicates that endpoint ratio changes are primarily driven by altered phototactic responses. Definitive mechanistic conclusions would require future single-cell trajectory tracking under controlled light gradients.

#### 2.2.3. Determination of Flagellar Shedding Rate

The flagellar shedding of *E. gracilis* cells was observed under a microscope, and the flagellar shedding rate was calculated. An aliquot of 20.0 μL of algal suspension was placed on a hemocytometer and allowed to settle for 5.0 min. A total of 300 cells were randomly counted to determine the flagellar shedding rate. Each sample was measured in triplicate, and three independent parallel experiments were performed.

### 2.3. Growth Curve and Photosynthetic Activity Measurement

*E. gracilis* cells at the logarithmic growth phase were collected and resuspended in freshly prepared culture medium to adjust the optical density at 680 nm (OD_680_) to 0.1. The cells were then exposed to PFOS at concentrations of 0, 0.5, 5.0, 50.0, and 500.0 μg/L. Samples were taken every 24 h over a period of 10 days to determine cell density. The 500 µg/L group was included to simulate extreme exposure scenarios (e.g., industrial accidental spills) and to capture the full dose–response range for growth inhibition. The maximum photochemical quantum yield of photosystem II (*Fv*/*Fm*) and non-photochemical quenching (*NPQ*) were measured daily using an AquaPen-C AP 110-C pulse-amplitude-modulated fluorometer (Photon Systems Instruments, Czech Republic) after a 15-min dark adaptation period. The instrument settings were as follows: measuring light at 30% of maximum output; saturating pulse intensity at 70% (~2100 µmol·m^−2^·s^−1^); actinic light at 300 µmol·m^−2^·s^−1^ (consistent with culture illumination). After dark adaptation, *Fv*/*Fm* was determined by a saturating pulse. *NPQ* was acquired after 5–10 min of actinic light adaptation under the same actinic light intensity. All measurements were conducted at OD_680_ = 0.15–0.30; within this range, no signal overflow was detected, and validation experiments confirmed that *Fv*/*Fm* and *NPQ* values remain stable and are unaffected by biomass concentration. Each sample was measured with three technical replicates; means were used for statistical analysis. It should be noted that *Fv/Fm* reflects only the maximum photochemical efficiency of PSII and cannot be extrapolated to represent overall photosynthesis; all discussion of these parameters is strictly limited to the PSII level.

### 2.4. Chlorophyll Extraction and Quantification

Aliquots of 10 mL of cell suspension cultured for 7 days were filtered and rinsed three times with distilled water. The cells were then ground with glass sand and 80% (*v*/*v*) acetone solution until the pigments were completely transferred to the solvent. The homogenate was filtered again to remove insoluble residues. The filtrate was collected and brought to a final volume of 10 mL with 80% acetone. The absorbance of the extract was measured at 470, 646, and 663 nm using a UV–Vis spectrophotometer [14]. Photosynthetic pigment concentrations (mg·L^−1^) were calculated according to the following equations:

$$C_{a}\;=\;12.21{\mathrm{Abs}}_{663}-2.81{\mathrm{Abs}}_{646}$$

$$C_{b}=20.13{\mathrm{Abs}}_{646}-5.03{\mathrm{Abs}}_{663}$$

$$C_{x+c}=(1000{\mathrm{Abs}}_{470}-3.27{\mathrm{Chl}}_{a}-104{\mathrm{Chl}}_{b})/229$$

where *C_a_*, *C_b_* and *C_x_*_+*c*_ are Chlorophyll a (Chl_a_), Chlorophyll b (Chl_b_), carotenoids and other pigments (*C_x_*_+*c*_), respectively.

### 2.5. Intracellular ATP Level Measurement

Intracellular ATP levels in *E. gracilis* were determined using an ATP Assay Kit (Beyotime Biotechnology, Shanghai, China). After PFOS exposure, cells were collected by centrifugation at 3000 rpm. The cell pellet was resuspended in 200.0 μL of lysis buffer and vortexed. Following lysis, the mixture was centrifuged at 12,000× *g* for 5 min at 4 °C. The supernatant (100.0 μL) was collected, and the relative luminescence unit (RLU) was measured using a multimode microplate reader (BioTek Instruments, Inc., Winooski, VT, USA).

### 2.6. Intracellular Reactive Oxygen Species (ROS) Level Measurement

Intracellular ROS levels were measured using a Reactive Oxygen Species Assay Kit (Beyotime Biotechnology, Shanghai, China). After PFOS exposure, cells were collected by centrifugation at 3000 rpm. The cell pellet was resuspended in phosphate-buffered saline (PBS) containing 2′,7′-dichlorodihydrofluorescein diacetate (DCFH-DA) diluted 1:2000 (*v*/*v*) in PBS. The suspension was incubated at 37 °C for 20 min. The cells were then washed three times with serum-free culture medium. Cells treated with the ROS positive control provided in the kit served as the control group. The stained cells were analyzed using a flow cytometer with excitation at 488 nm and emission at 525 nm. A total of 12,000 events were recorded per sample.

### 2.7. Assays of Isolated Chloroplast Function

Chloroplasts were isolated from *E. gracilis* cells cultured under autotrophic conditions for 10 days using a High-Purity Chloroplast Isolation Kit (density gradient method, Beyotime Biotechnology, Shanghai, China), and the isolated chloroplasts were characterized by fluorescence microscopy to confirm morphological integrity (see Figure S3 for representative optical and fluorescence microscopy images of isolated chloroplasts). The bioaccumulation of organic pollutants in the environment is influenced by multiple factors, including subcellular distribution, the specific surface area of organisms, and environmental concentrations [24,25]. Studies have shown that PFOS concentrations at the subcellular level typically range from pg/g to μg/g, generally not exceeding 10 ng/g, although levels may exceed this value in tissues or organelles prone to PFOS accumulation [26,27]. Based on these literature estimates of subcellular PFOS accumulation levels, isolated chloroplasts were exposed to PFOS at concentrations of 0, 5.0, 50.0, and 500.0 ng/L as experimental approximation concentrations for in vitro chloroplast exposure. It should be noted that PFOS concentrations in isolated *Euglena* chloroplasts were not directly measured; the correspondence between external exposure concentrations and actual intra-chloroplast concentrations remains a theoretical inference. These concentrations are therefore not defined as actual subcellular accumulation levels but rather as an experimental approximation range for assessing the directaction potential of PFOS on chloroplasts.

#### 2.7.1. Photophosphorylation Assay

An aliquot of 0.1 mL of chloroplast suspension was mixed with 0.9 mL of reaction buffer containing 0.015 mol/L Tris-HCl, 0.035 mol/L NaCl, 0.01 mmol/L MgCl_2_, 1 µmol/L ATP (added as a carrier to stabilize the endogenous ATP pool of isolated chloroplasts, preventing kinetic artifacts from low background ATP), and 1 μmol/L K_3_Fe(CN)_6_ (as the terminal electron acceptor for photosynthetic electron transport). The mixture was illuminated at a PPFD of approximately 52.5 µmol·m^−2^·s^−1^ (3500 lux) for 1 min at 25 °C. Inorganic phosphorus content was then determined using a commercial phosphate assay kit (Beyotime Biotechnology, Shanghai, China). Under illumination, photosynthetic electron transport reduces ferricyanide, establishing a proton gradient across the thylakoid membrane that drives ATP synthesis from endogenous ADP and free Pi. Consequently, the decrease in free Pi after illumination reflects net photophosphorylation activity. It should be noted that this method can only semi-quantitatively characterize photophosphorylation activity and is not suitable for precise absolute ATP synthesis rate determination. Independent dark controls were not included, which constitutes a limitation; all treatment groups were measured under identical illumination and Pi determination conditions, so relative Pi changes among groups remain comparable. Boiled chloroplast controls and uncoupler-treated controls were not performed. Results are presented for relative comparison among PFOS treatment groups only.

#### 2.7.2. Mg^2+^-ATPase Activity Assay

An aliquot of 0.1 mL of chloroplast suspension was mixed with 0.5 mL of activation buffer containing 25 mmol/L Tris-HCl, 50.0 mmol/L NaCl, 5.0 mmol/L MgCl_2_, 1 μmol/L EDTA, and 200.0 μg trypsin (trypsin pretreatment removes the inhibitory ε-subunit of ATP synthase, enabling synthase operation in the hydrolytic direction). The mixture was illuminated at 25 °C for 5 min for activation, and the reaction was terminated by adding 0.1 mL of bovine serum albumin (5 mg/mL). Subsequently, 0.5 mL of reaction buffer containing 50.0 mmol/L Tris-HCl, 5.0 mmol/L MgCl_2_, and 5.0 μmol/L ATP was added. The mixture was incubated in a water bath at 37 °C for 10 min, and the reaction was terminated by adding 0.1 mL of 20% trichloroacetic acid. After centrifugation, the supernatant was collected to determine ATP hydrolysis-derived inorganic phosphorus. This assay measures the total Mg^2+^-dependent ATP hydrolytic activity of the chloroplast-enriched fraction, not the specific activity of CF_1_CF_0_-ATP synthase alone. Three limitations are acknowledged: (i) potential PFOS interference with the phosphate colorimetric detection system was not independently verified; (ii) inhibitor controls were not performed (e.g., oligomycin for mitochondrial ATPase, sodium vanadate for plasma membrane ATPase); (iii) although chloroplast morphological integrity was confirmed by fluorescence microscopy, purity was not validated using organelle-specific marker enzymes. Accordingly, results are interpreted as reflecting Mg^2+^-dependent ATP hydrolytic activity in the chloroplast-enriched fraction, and mechanistic conclusions regarding specific ATP synthase targeting are presented as hypotheses rather than definitive findings.

#### 2.7.3. Ca^2+^-ATPase Activity Assay

An aliquot of 0.1 mL of chloroplast suspension was mixed with 0.5 mL of activation buffer containing 25 mmol/L Tris-HCl, 2 µmol/L EDTA (to chelate endogenous Mg^2+^ and suppress Mg^2+^-dependent ATPase activities), and 1 μmol/L ATP. The mixture was illuminated at 25 °C for 5 min for activation, and the reaction was terminated by adding 0.1 mL of bovine serum albumin (5.0 mg/mL). Subsequently, 0.5 mL of reaction buffer containing 50 mmol/L Tris-HCl, 2 mmol/L CaCl_2_ (as the sole divalent cation), and 10 μmol/L ATP was added. The mixture was incubated in a water bath at 37 °C for 10 min, and the reaction was terminated by adding 0.1 mL of 20% trichloroacetic acid. After centrifugation, the supernatant was collected to determine ATP hydrolysis-derived inorganic phosphorus. Under these conditions, the measured ATP hydrolytic activity derives from Ca^2+^-dependent ATPases in the chloroplast-enriched fraction. It should be noted that the density-gradient-prepared chloroplast fraction may contain multiple Ca^2+^-dependent ATPases (e.g., ER SERCA-type calcium pumps and residual plasma membrane Ca^2+^-ATPase), in addition to chloroplast CF_1_CF_0_-ATP synthase (whose Ca^2+^-dependent activity is far lower than its Mg^2+^-dependent activity). Therefore, results from this crude enzyme system cannot directly demonstrate specific targeting of chloroplast ATP synthase by PFOS. The core conclusion relies on the differential response between Mg^2+^-dependent and Ca^2+^-dependent activities.

#### 2.7.4. Cytochrome b_6_f Complex Activity Assay

Chloroplasts were isolated and suspended in a buffer containing 50 mM K_2_HPO_4_ (pH 6.5), 0.33 M sorbitol, 1.0 mM MgCl_2_, 1.0 mM MnCl_2_, 2.0 mM EDTA, and 1% Triton X-100. The chloroplast suspension was exposed to PFOS at concentrations of 0.0, 5.0, 50.0, and 500.0 ng/L, and the absorbance peak at 554 nm was measured [28].

#### 2.7.5. Electron Transport Rate Assay

Isolated chloroplasts were resuspended in Tris-HCl buffer and adjusted to a concentration of 50.0–100.0 μg chlorophyll/mL. Chlorophyll content was determined using a spectrophotometer at 663 nm and 645 nm, and the concentration was further diluted to the desired range for the experiment. The artificial electron acceptor DCIP (2,6-dichlorophenolindophenol) was freshly prepared at a final concentration of 50 µmol/L and protected from light to prevent decomposition. Buffer without chloroplasts was added to a cuvette, and the initial absorbance of DCIP at 600 nm was recorded. Chloroplast suspensions containing PFOS at concentrations of 0.0, 5.0, 50.0, and 500.0 ng/L were then mixed with the DCIP solution. The reaction was allowed to proceed under illumination at a PPFD of approximately 75 µmol·m^−2^·s^−1^ (5000 lux) for 2 min, and the change in absorbance was recorded [29]. All ETR values were normalized to chlorophyll content. The electron transport rate is expressed as the change in absorbance at 600 nm per unit time (ΔAbs_600_/min), reflecting the rate of DCIP photoreduction by the photosynthetic electron transport chain.

#### 2.7.6. Cyclic Voltammetry (CV) Measurements

Cyclic voltammetry experiments were conducted in 40.0 mL of solution using a three-electrode system consisting of a glassy carbon working electrode (3.0 mm diameter), a platinum wire counter electrode, and an Ag/AgCl reference electrode. The voltage was scanned from −0.8 V to 1.0 V (vs. Ag/AgCl) at a scan rate of 0.1 V/s.

#### 2.7.7. Electrochemical Impedance Spectroscopy (EIS) Measurements

Electrochemical impedance spectroscopy (EIS) was performed to probe the electrical properties of the chloroplast/electrode interface, and was performed under potentiostatic control at the open-circuit potential (OCP) at room temperature. The electrochemical test system operated over a frequency range of 10^5^ to 10^−1^ Hz. A standard three-electrode system was employed, with a glassy carbon electrode serving as the working electrode, and an Ag/AgCl electrode and a platinum wire as the reference and counter electrodes, respectively. To quantitatively analyze the impedance spectra, the equivalent circuit model shown in Figure S4 was employed. The high-frequency semicircle is attributed to the charge transfer process at the electrode–chloroplast interface, and its diameter corresponds to *R*_2_ (charge transfer resistance), reflecting the efficiency of electron transfer from the photosynthetic electron transport chain to the electrode. The intermediate-frequency semicircle is assigned to the resistance of the thylakoid membrane or the biointerface (*R*_1_), which is sensitive to the integrity and ion permeability of the membrane. The low-frequency linear region (with a slope approaching 45°) is characteristic of the Warburg impedance (*Z*_0_), indicating that the overall reaction becomes limited by the diffusion of redox species (e.g., electron carriers or ions) near the electrode surface.

### 2.8. Transcriptomic and Proteomic Analyses

#### 2.8.1. Transcriptome Analysis

*E. gracilis* cells exposed to 0.0 or 50.0 μg/L PFOS for 7 days (three biological replicates per group) were harvested for transcriptome analysis. Total RNA was sequenced using the Illumina platform (non-reference approach). Raw reads were filtered to obtain clean reads, which were then de novo assembled into unigenes. The unigenes served as reference sequences for expression quantification, functional annotation, and identification of SNPs and SSRs [30]. Differentially expressed genes (DEGs) were screened with |log_2_ fold change| > 2 and *p* < 0.05. Quality control metrics included raw reads (>10 million), raw bases (>100 Mb), clean reads (>70% of raw reads), clean bases (>80 Mb), error rate (<1%), Q20 (≥90%), Q30 (≥85%), and GC content (approximately 62%, within the acceptable range for microalgae [31,32]). All metrics met the standard requirements for reliable transcriptomic analysis. Gene functional annotation was performed using NCBI, UniProt, KEGG, and GO public databases.

#### 2.8.2. Quantitative Proteome Analysis

Protein extracts from the same samples (0.0 and 50.0 μg/L PFOS, 7 days) were analyzed using data-independent acquisition (DIA) mass spectrometry [33]. A protein database was constructed based on the de novo transcriptome assembly of *E. gracilis*. DIA data were processed for protein identification and quantification. Differentially expressed proteins (DEPs) were screened with fold change ≥ 1.2 and *p* < 0.05; the Benjamini–Hochberg procedure was applied for multiple-testing correction to control the false discovery rate. Quality control was assessed by monitoring peptide retention time (*RT*) consistency across samples and the proportion of unique peptides. Peptides with coefficient of variation (*CV*) < 0.3 and present in all samples were used as internal standards. The majority of identified proteins contained multiple unique peptides, confirming the reliability of the proteomic data [34]. Protein functional annotation was performed using NCBI, UniProt, KEGG, and GO public databases.

### 2.9. Statistical Analysis

All experiments were conducted with three independent biological replicates, where each replicate originated from a separate algal culture batch. Data are presented as the arithmetic mean ± standard deviation (s.d.). Experimental data were processed using Excel 2022. Graphs were created with Origin 2024. Statistical analyses were performed using IBM SPSS Statistics 27. When evaluating the effect of PFOS concentration, data at each individual time point were independently analyzed by one-way analysis of variance (ANOVA) followed by Fisher’s least significant difference (LSD) or Tukey’s HSD post hoc test. Significant differences among groups are indicated by different lowercase letters (a, b, c, d), with groups sharing the same letter showing no significant difference, and groups with different letters showing significant differences (*p* < 0.05).

## 3. Results and Discussion

### 3.1. PFOS Accumulation in the Eyespot Impairs Phototaxis and Raises Light Threshold

**Figure 2:**
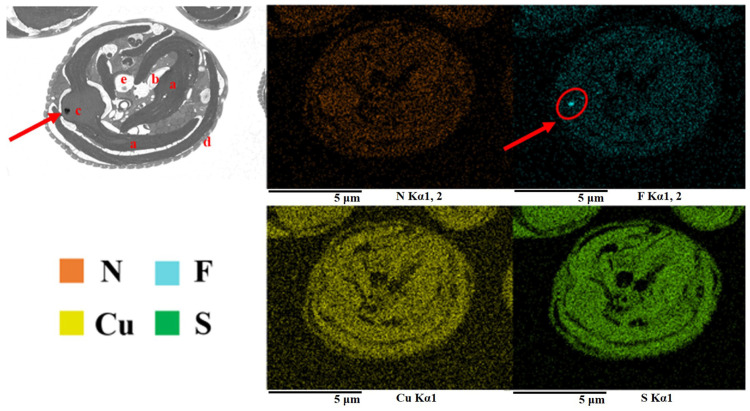
Subcellular distribution of PFOS in *E. gracilis* visualized by transmission electron microscopy (TEM) and corresponding TEM-energy-dispersive X-ray spectroscopy (EDS) mapping. Red arrow points to the eyespot. (**top left**): TEM morphology image; (**bottom left** and **right panels**): EDS elemental distribution maps. TEM images of cell sections with major organelles labeled: chloroplast (a), paramylon body (b), eyespot (c), plasma membrane (d), and vacuole (e). The enhanced fluorine signals (indicative of PFOS accumulation) are observed in the eyespot.

To investigate the subcellular distribution of PFOS and its relationship with toxic phenotypes, transmission electron microscopy combined with energy-dispersive X-ray spectroscopy mapping (TEM-EDS mapping) was performed on *E. gracilis* cells exposed to 50.0 μg/L PFOS for 7 days. As shown in Figure 2, fluorine signal enrichment exhibited a pronounced pattern: stronger fluorine signals, indicative of PFOS accumulation, were detected in the eyespot (the highest) and chloroplasts, whereas almost no enrichment was observed in vacuoles or paramylon bodies. As no fluorine-containing components were present in the culture medium, reagents, or embedding resin, the detected fluorine signal can be attributed to PFOS. It should be noted that TEM-EDS mapping provides qualitative characterization of fluorine spatial distribution and relative enrichment patterns, and does not yield quantitative subcellular PFOS concentrations. This distribution pattern suggests that, upon entering the cell, PFOS tends to accumulate in the eyespot, chloroplasts, and mitochondria. The high enrichment in the eyespot is particularly noteworthy, as the eyespot is located in the anterior plasma membrane region and may be more accessible to exogenous PFOS. Moreover, the carotenoid–protein complexes abundant in the eyespot may bind PFOS through hydrogen bonds and intermolecular forces [35], potentially disrupting the complex structure and interfering with phototransduction. Meanwhile, fluorine signal enrichment in chloroplasts suggests PFOS may disturb thylakoid membrane structure and electron transport chain function, while its presence in mitochondria may affect the tricarboxylic acid (TCA) cycle and oxidative phosphorylation.

**Figure 3:**
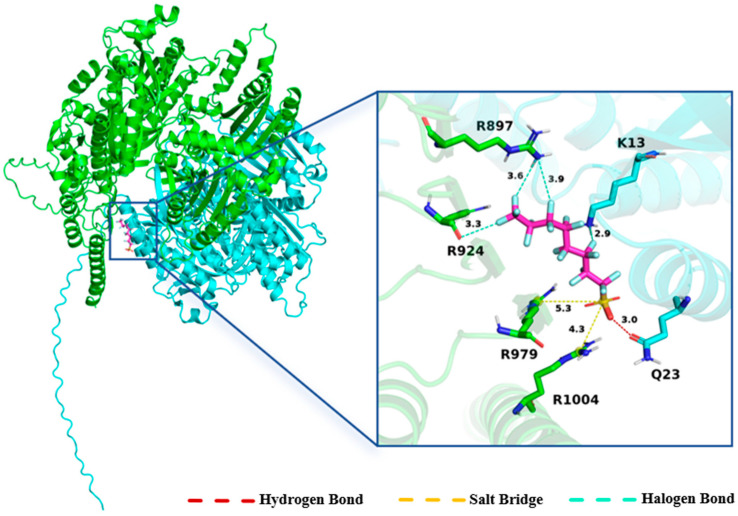
Molecular docking of PFOS with PAC. PFOS (pink stick, with wathet fluorine atoms) is predicted to bind to the active pocket of PAC. Hydrogen/halogen bonds and salt bridges are indicated by dashed lines.

To computationally assess whether PFOS can interact with the photoreceptor protein in the eyespot, the photoactivated adenylyl cyclase (PAC), which is involved in blue-light signal transduction, was selected as the docking target. As a high-resolution crystal structure of PAC is currently unavailable, a predicted structure was obtained by homology modeling to evaluate the binding potential of PFOS to the active pocket. The docking results (Figure 3) showed a predicted binding energy of −9.2 kcal/mol between PFOS and PAC. This value falls within the reported energy range for potential stable PFOS–target protein complexes (−5.2 to −9.7 kcal/mol) [36], suggesting a theoretical potential binding trend. PFOS was predicted to localize within the active pocket and potentially form hydrogen bonds, halogen bonds, or salt bridges with residues such as Arg126, Tyr204, and Trp98. This is consistent with the general propensity of PFOS to undergo electrostatic interactions with basic amino acid residues of various proteins [37]. It must be emphasized that, in the absence of an experimentally resolved PAC structure, these molecular docking results are purely theoretical predictions and can only serve to generate scientific hypotheses; they are insufficient to confirm that PFOS binds PAC in vivo or inhibits PAC function. Functional validation through measurement of light-induced cAMP production, PAC-dependent signaling, and phototactic responses under defined blue-light stimulation will be the focus of our subsequent investigations.

**Figure 4:**
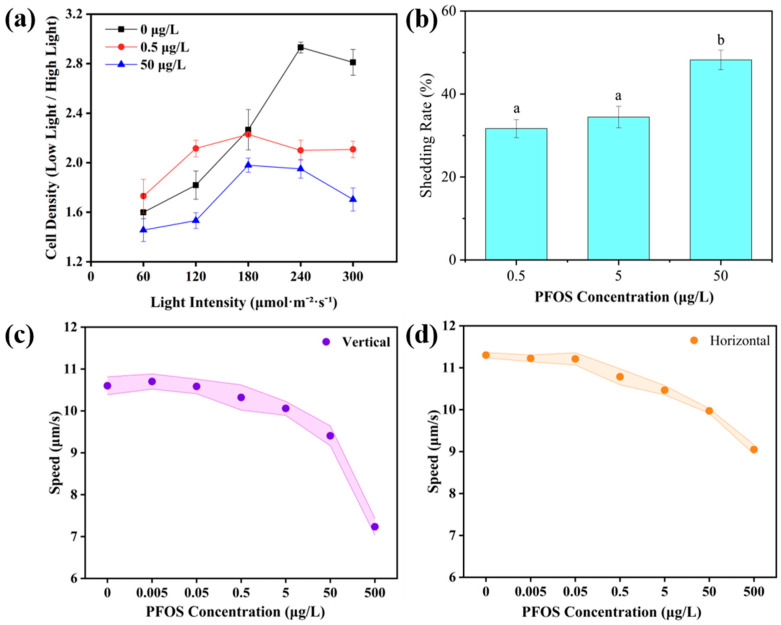
Effects of PFOS exposure on phototaxis, flagellar shedding, and apparent migration velocity of *E. gracilis*. (**a**) Phototactic response (ratio of cell density at the near-light end to that at the far-light end) under different light intensity. (**b**) Flagellar shedding rate. (**c**) Vertical apparent migration velocity. (**d**) Horizontal apparent migration velocity. Different lowercase letters indicate significant differences among groups (*p* < 0.05).

Phototaxis assays further revealed changes in the phototactic behavior of *E. gracilis* after PFOS exposure (Figure 4a). At a low PFOS concentration (0.5 μg/L), an altered phototactic response was observed: at low light intensities (PPFD ~60–120 µmol·m^−2^·s^−1^, corresponding to 2000–4000 lux), the sensitivity of cells to light was markedly enhanced; however, when the light intensity increased to PPFD ~180–300 µmol·m^−2^·s^−1^ (6000–10,000 lux), the phototactic response diminished, and the light saturation threshold decreased from PPFD ~300 µmol·m^−2^·s^−1^ (10,000 lux) (control) to approximately PPFD ~180 µmol·m^−2^·s^−1^ (6000 lux). A possible explanation, which remains a hypothesis requiring functional validation, is that mild binding of PFOS to PAC at low concentrations may lower the trigger threshold for phototransduction, resulting in enhanced sensitivity hypersensitivity under weak light. As light intensity increases, the impaired light-adaptation feedback mechanism fails to properly regulate signal gain, leading to premature light saturation [38]. At a high PFOS concentration (50.0 μg/L), global phototaxis inhibition occurred, with the entire light-response curve shifted downward, indicating severe functional damage to the photoreceptive system. It should be noted that the endpoint ratio method used cannot completely exclude potential interference from reduced cell motility; however, the gradient-dependent nature of the response (enhanced sensitivity at low light vs. premature saturation at high light, rather than uniform suppression across all intensities) and corroborating TEM-EDS evidence of eyespot fluorine enrichment collectively support that the observed changes are primarily driven by altered phototactic responses, rather than by nonspecific motility impairment alone.

Flagella are the executive organs of phototactic movement. The flagellar shedding rate increased in a concentration-dependent manner after PFOS exposure (Figure 4b): at low concentrations (0.5–5.0 μg/L), the shedding rate increased slightly but not significantly, whereas at 50.0 μg/L it rose markedly from 30% to 48.7%, and microscopic observation revealed evident flagellar shedding in PFOS-treated cells (Figure S5). This change directly impairs active cell motility. Apparent migration velocity measurements (Figure 4c,d) were consistent with this pattern: both vertical and horizontal Apparent migration velocity decreased with increasing PFOS concentration, with vertical movement being more severely affected. The increase in flagellar shedding may be related to PFOS-induced disruption of membrane lipid–protein interactions [39], and the reduced vertical migration ability may further affect the vertical migration of cells in the water column and their access to light resources. The apparent migration velocities reported here (range ~7–11 µm/s) represent population-level net migration through the chamber over 20 min, which is a distinct parameter from single-cell instantaneous swimming speed (typically tens to >100 µm/s for Euglena). These values are consistent with the reported range of Euglena population apparent migration velocities in the literature (5.3–33.3 µm/s) [40,41].

In summary, the eyespot accumulation and phototactic abnormalities observed in this section reflect a direct disturbance of the photoreceptive-motor system by PFOS. This disturbance impairs the ability of *E. gracilis* to actively approach or escape from specific light environments, thereby compromising the spatial positioning efficiency for light resource acquisition in the water column.

### 3.2. PFOS Decouples Electron Transport from ATP Synthesis, Reducing ETR and ATP While Increasing NPQ

Although PFOS accumulated markedly in the eyespot and impaired cell motility, PSII photochemical efficiency was not suppressed in the short term; instead, a moderate enhancement was observed. *E. gracilis* contains chlorophyll a (Chl a), chlorophyll b (Chl b), carotenoids and other pigments (Cx + c). As shown in Figure S6, exposure to 0.5 and 50.0 μg/L PFOS generally increased intracellular chlorophyll content, with Chl a being the most responsive. Compared with the control, 0.5 μg/L PFOS significantly elevated Chl a content; Chl b and other pigments also increased, though not significantly. At 50.0 μg/L PFOS, chlorophyll content still tended to be higher than the control, but the difference did not reach statistical significance. Overall, environmentally relevant PFOS caused a slight stimulation of chlorophyll content, which is more likely a short-term stress response rather than a genuine improvement in photosynthetic capacity. Similar inductions of photosynthetic pigments have been reported under other pollutant stresses [42] and are generally interpreted as a compensatory strategy when light capture efficiency is threatened.

**Figure 5:**
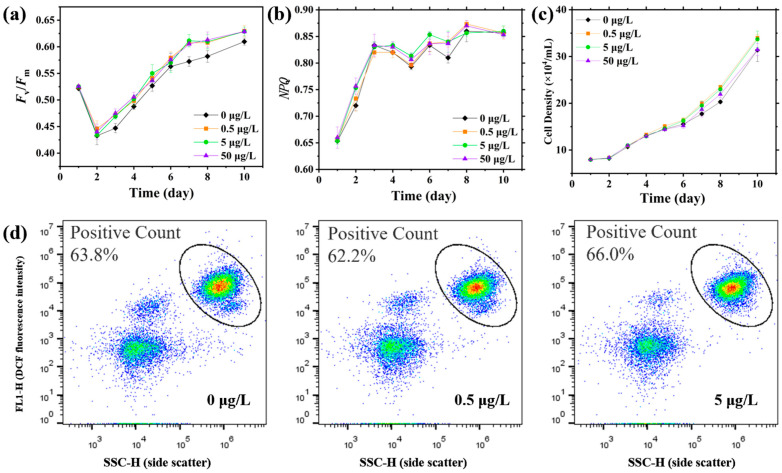
Effects of PFOS exposure on PSII parameters, growth, and ROS levels in *E. gracilis*. (**a**) Maximum photochemical efficiency of PSII (*Fv*/*Fm*). (**b**) Non-photochemical quenching (*NPQ*). (**c**) Growth curves (cell density over 10 days). (**d**) Intracellular reactive oxygen species (ROS) levels (percentage of cells with high ROS positivity). Flow cytometric analysis of intracellular ROS levels using DCFH-DA (excitation 488 nm, emission 525 nm). *X*-axis: DCF fluorescence intensity (FL1-H, reflecting ROS level); *Y*-axis: side scatter (SSC-H, reflecting cell granularity). Gates indicate the percentage of cells with high ROS positivity. A total of 12,000 events were recorded per sample.

Non-photochemical quenching (*NPQ*) is a key photoprotective mechanism that dissipates excess excitation energy as heat, whereas *Fv*/*Fm* reflects the maximum photochemical efficiency of photosystem II (PSII). Under normal conditions, increased *NPQ* indicates enhanced photoprotection, and decreased *Fv*/*Fm* signifies damage to PSII reaction centers. Previous studies have shown that high PFOS concentrations damage microalgal photosynthesis, while lower concentrations may cause transient stimulation [43]. In this study, exposure to 0.5–50.0 μg/L PFOS led to significant increases in *Fv*/*Fm* at several time points, particularly on day 3, and the elevation became even more pronounced by day 7 (Figure 5a). *NPQ* also increased significantly on days 2 and 7 (Figure 5b). The temporal patterns of *Fv*/*Fm* and *NPQ* were generally consistent, suggesting that PFOS triggered a photoprotective regulatory response. Zheng et al. [44] also observed a short-term stimulatory effect of low-concentration PFOS (≤5.0 µg L^−1^) on the growth of the marine diatom *Thalassiosira minima* Gaarder. Nevertheless, such upregulation should be viewed as an adaptive stress response rather than evidence of true photosynthetic improvement. It is important to note that *Fv*/*Fm* reflects only the maximum photochemical efficiency of PSII and does not represent overall photosynthesis; the discussion of these parameters is therefore limited to the PSII level.

Importantly, the simultaneous increases in chlorophyll content, *Fv*/*Fm*, and *NPQ* are not readily reconciled with the expectation that eyespot damage would reduce light capture capacity. In other words, although PFOS accumulated in the eyespot and impaired phototaxis, the in vivo PSII photosynthetic system did not show a corresponding downregulation; instead, it displayed signs of upregulation. This discrepancy suggests that eyespot dysfunction does not directly translate into a measurable loss of light capture efficiency under the present experimental conditions, and the observed changes in PSII parameters are more likely attributable to direct effects of PFOS on chloroplasts. It should be noted that oxygen evolution was not directly measured in this study; the observed changes in chlorophyll content, *Fv*/*Fm*, and photosynthesis-related gene expression do not necessarily reflect net O_2_ production rates. Direct measurement of oxygen evolution would be a valuable addition to future investigations.

Does this short-term upregulation of photosynthetic parameters confer a real growth advantage? Growth curve monitoring (Figure 5c) revealed that low PFOS concentrations (0.5–5.0 μg/L) promoted growth on days 7–10, with the 0.5 μg/L group showing the strongest effect. The 50.0 μg/L group also exhibited a weak promotion on days 7–8, but by day 10, it began to show an inhibitory trend, with cell density falling below that of the control. Thus, the effect of PFOS on growth is not a simple linear inhibition. At early time points and lower concentrations, cells may obtain a transient growth stimulus via compensatory mechanisms; however, as exposure time and concentration increase, toxicity accumulates and eventually dominates. This low-concentration stimulation/high-concentration inhibition pattern is known as hormesis [45], which typically reflects a dynamic balance between the activation and exhaustion of cellular defense systems.

**Figure 6:**
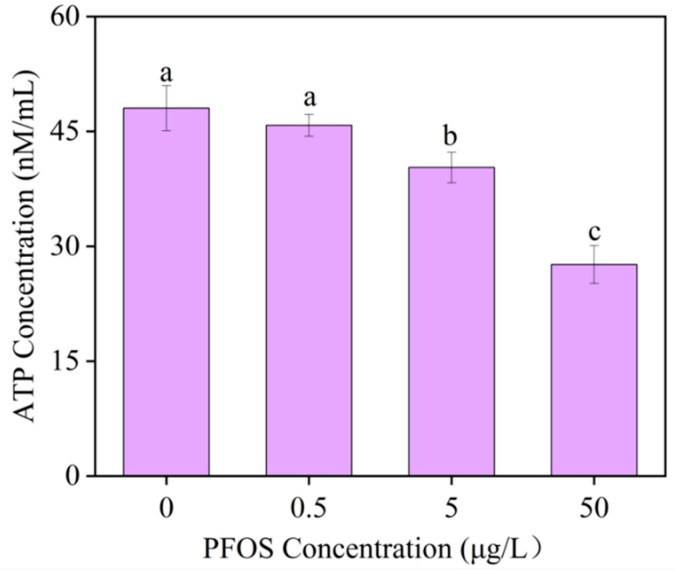
Intracellular ATP levels in *E. gracilis* exposed to different concentrations of PFOS (0, 0.5, 5.0, and 50.0 μg/L). Data are presented as mean ± SD (*n* = 3). Different lowercase letters indicate significant differences among groups (*p* < 0.05).

Nevertheless, the weak growth promotion did not translate into improved cellular energy status. Further analysis showed that intracellular ATP levels decreased progressively with increasing PFOS concentration (Figure 6). Compared with the control, ATP content in all treatment groups was significantly reduced in a concentration-dependent manner. Hence, although some PSII parameters were upregulated, energy metabolism did not improve; on the contrary, energy output appeared constrained. This indicates that the primary impact of PFOS on *E. gracilis* may not be on light capture per se, but rather on downstream energy conversion processes. Measurements of reactive oxygen species (ROS) provide supporting evidence. PFOS exposure elevated intracellular ROS levels (Figure 5d). Relative to the control, the proportion of cells with high ROS positivity decreased slightly from 63.8% to 62.2% at 0.5 μg/L PFOS but increased to 66% at 5.0 μg/L PFOS. This pattern is consistent with a hormetic response: at low concentrations, the antioxidant defense system may be moderately activated, preventing a rise in ROS; at higher concentrations, oxidative stress overwhelms the scavenging capacity, leading to ROS accumulation. Elevated ROS can directly damage photosynthetic membrane lipids and protein complexes and also consume cellular reducing equivalents, further burdening the electron transport chain.

Taken together, the in vivo data in this section reveal a coherent yet paradoxical picture: after PFOS exposure, PSII parameters (chlorophyll, *Fv*/*Fm*, *NPQ*) and growth show transient upregulation, but ATP output continuously declines, and ROS levels display a concentration-dependent biphasic pattern. This decoupling between the PSII phenotype and energy metabolism—especially the divergence between elevated *Fv*/*Fm* and reduced ATP—strongly suggests that direct interference of PFOS with chloroplast energy conversion (rather than an initial defect in light capture) is the key to understanding these seemingly contradictory observations.

### 3.3. PFOS Inhibits Mg^2+^-Dependent ATP Hydrolytic Activity in the Chloroplast-Enriched Fraction, Accompanied by Reduced Electron Transport

The in vivo observations of “enhanced light capture but decreased ATP output” together with increased ROS levels suggested that PFOS may affect the photosynthetic system of *E. gracilis* through two possible pathways: (i) indirect effects via accumulation in the eyespot and flagella, which might disturb whole-cell physiology, and (ii) direct actions on chloroplasts, such as inhibition of ATP synthase or interference with the electron transport chain. To distinguish between these possibilities, isolated chloroplasts from autotrophically grown *E. gracilis* were exposed to PFOS concentrations (0–500 ng/L) as an experimental approximation of subcellular accumulation levels.

**Figure 7:**
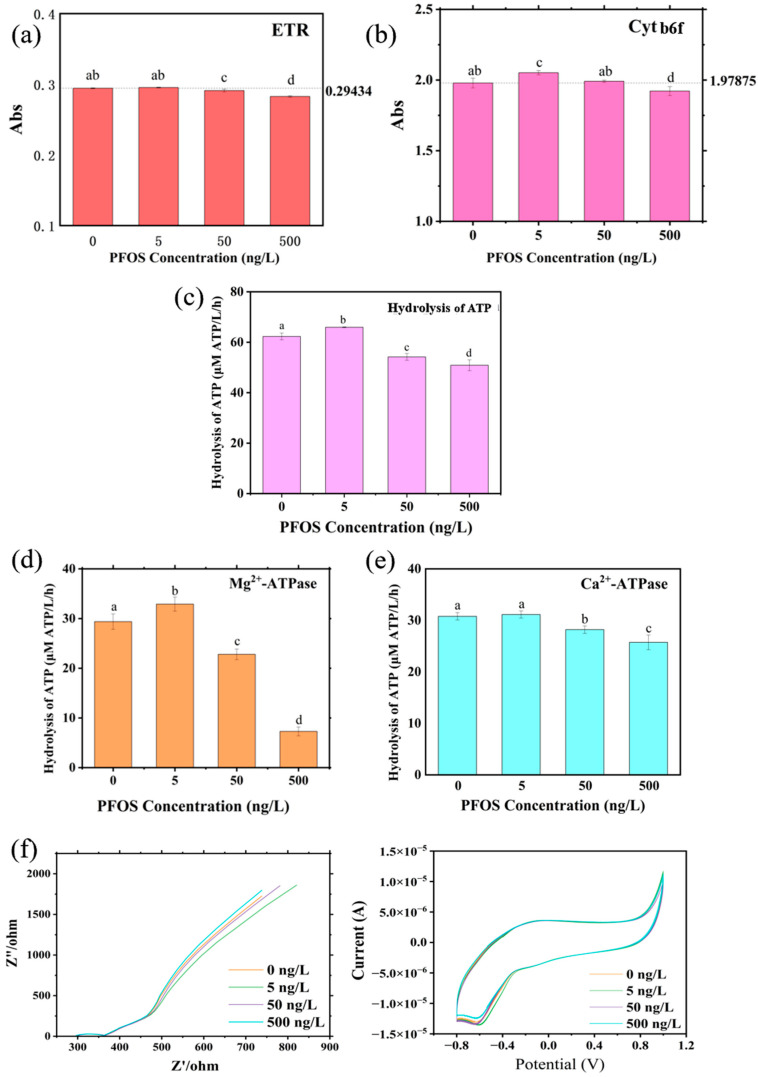
Effects of PFOS on isolated chloroplasts of *E. gracilis*. (**a**) Photosynthetic electron transport rate (*ETR*, expressed as ΔAbs_600_/min). (**b**) Cytochrome b6f complex activity. (**c**) Photophosphorylation activity. (**d**) Mg^2+^-dependent ATP hydrolytic activity. (**e**) Ca^2+^-dependent ATP hydrolytic activity. (**f**) Electrochemical impedance spectroscopy (EIS) Nyquist plots and cyclic voltammetry (CV) curves. Chloroplasts were exposed to PFOS at 0, 5.0, 50.0, and 500.0 ng/L. Data in (**a**–**e**) are presented as mean ± SD (*n* = 3). Different lowercase letters indicate significant differences among groups (*p* < 0.05).

The effect of PFOS on the photosynthetic electron transport rate (*ETR*) of isolated chloroplasts was concentration-dependent. No significant change in *ETR* was observed at 5.0 ng/L PFOS, whereas *ETR* decreased significantly at 50.0 and 500.0 ng/L (*p* < 0.05; Figure 7a). *ETR* reflects the overall operation of the photosynthetic electron transport chain. Comparing the responses of ETR and Cyt b_6_f revealed that *ETR* was much more sensitive to PFOS than Cyt b_6_f. At 50.0 ng/L, Cyt b_6_f decreased only mildly, whereas *ETR* was already significantly inhibited. This discrepancy suggests that the suppression of electron transport is unlikely to be explained solely by direct inhibition of a single complex such as Cyt b_6_f. Consistent with the ATP synthase activity data below, one plausible interpretation is that the decrease in *ETR* is secondary to impaired chloroplast ATP synthase function, rather than resulting solely from direct damage to electron transport chain components. However, this mechanistic interpretation remains a hypothesis; direct validation would require additional experiments using uncouplers (e.g., nigericin or gramicidin) and comparison of phosphorylating versus non-phosphorylating conditions, and direct measurement of the trans-thylakoid proton gradient.

The effect of PFOS on cytochrome b_6_f (Cyt b_6_f) complex activity was examined first (Figure 7b). Cyt b_6_f is a critical electron transfer node between photosystem II (PSII) and photosystem I (PSI). Cyt b_6_f activity increased slightly but not significantly at 5.0 ng/L PFOS, then gradually decreased at 50.0 and 500.0 ng/L, with significant inhibition observed only at 500.0 ng/L.

Impairment of the electron transport chain inevitably affects the establishment of the trans-thylakoid proton gradient, which is the direct driving force for ADP phosphorylation by ATP synthase [46]. Measurements of photophosphorylation activity (Figure 7c–e) provided evidence that ATP synthase function is impaired. At 5.0 ng/L PFOS, photophosphorylation activity increased slightly but not significantly; at 50.0 ng/L, activity decreased significantly (*p* < 0.05); and at 500.0 ng/L, the decrease was even more pronounced (*p* < 0.01). The change in Mg^2+^-ATPase activity (Figure 7d) followed a pattern similar to that of photophosphorylation, whereas Ca^2+^-ATPase activity (Figure 7e) did not change significantly in any treatment group. This ion-dependent selectivity suggests that the toxic effect is biased toward Mg^2+^-dependent enzymes, which is consistent with the strict Mg^2+^ dependence of chloroplast CF_1_CF_0_-ATP synthase. Desaiah [47] reported that certain organic pollutants can selectively inhibit Mg^2+^-ATPase while sparing Ca^2+^-ATPase activity, which is consistent with our findings. However, it must be noted that the Mg^2+^-ATPase assay measures total Mg^2+^-dependent ATP hydrolytic activity in the chloroplast-enriched fraction, not the specific activity of CF_1_CF_0_-ATP synthase alone, and inhibitor controls were not performed. The conclusion that PFOS impairs chloroplast ATP synthase function is therefore presented as a hypothesis, supported collectively by the decreased photophosphorylation, inhibited Mg^2+^-dependent (but not Ca^2+^-dependent) hydrolytic activity, reduced intracellular ATP, and proteomic downregulation of ATP synthesis-related proteins.

To further investigate the impact of PFOS on the electrochemical properties of chloroplasts, electrochemical impedance spectroscopy (EIS) and cyclic voltammetry (CV) were performed on isolated chloroplasts (Figure 7f). EIS results showed that the membrane/interface resistance in the equivalent circuit of chloroplasts first decreased and then increased with increasing PFOS concentration. At low concentration, a mild change in membrane fluidity might reduce ion transmembrane resistance; at high concentration, the interface membrane resistance increased significantly. This change likely does not arise from simple physical membrane damage but rather from multiple functional disturbances at the molecular level. The decline in ATP synthase activity leads to ATP shortage, which in turn impairs ATP-dependent transmembrane ion pumps (e.g., H^+^-ATPase, Ca^2+^-ATPase), further aggravating ion transport disturbance. This may be an important contributing factor to the increased membrane interface resistance. The CV curves showed that at 5.0 ng/L PFOS, both the oxidation and reduction peaks of chloroplasts were slightly enhanced, indicating that low-concentration PFOS might exert a mild stimulatory effect on the chloroplast membrane system, resulting in a transient increase in electrochemical response. At higher concentrations (50.0 and 500.0 ng/L), the oxidation and reduction peaks gradually weakened, suggesting that higher PFOS concentrations inhibit thylakoid membrane structure and electron transfer processes.

The key finding of the isolated chloroplast assays is that PFOS inhibits Mg^2+^-dependent ATP hydrolytic activity in the chloroplast-enriched fraction and impairs electron transport efficiency, even in the absence of whole-cell regulatory factors. The selective inhibition of Mg^2+^-dependent over Ca^2+^-dependent ATP hydrolytic activity is consistent with impaired chloroplast ATP synthase function, though the specific molecular target remains to be conclusively identified due to the lack of inhibitor controls and organelle-purity validation. This indicates that the action of PFOS on the photosynthetic apparatus has a degree of chloroplast autonomy and does not depend on eyespot signaling or nuclear regulation. The observed decrease in *ETR* may be secondary to impaired ATP synthase function; however, this mechanism remains to be directly validated.

Several methodological limitations of the isolated chloroplast assays should be explicitly noted. First, dark controls were not included in the photophosphorylation assay; all treatment groups were measured under identical illumination conditions, and relative Pi changes among groups are therefore comparable, but absolute light-driven versus dark ATP hydrolytic contributions cannot be distinguished. Second, boiled chloroplast controls, uncoupler treatments (e.g., nigericin or gramicidin), and phosphorylating versus non-phosphorylating conditions were not employed, which limits the ability to definitively establish whether *ETR* suppression results from chloroplast ATP synthase functional impairment or from direct damage to electron transport chain components. Third, organelle-purity markers were not assessed; although chloroplast morphological integrity was confirmed by fluorescence microscopy (Figure S3), the potential contribution of mitochondrial or plasma membrane ATPases to the measured Mg^2+^-ATP hydrolytic activity cannot be excluded. Fourth, potential interference of PFOS with the phosphate colorimetric detection system was not independently verified. Finally, inhibitor controls (e.g., oligomycin for mitochondrial ATPase, sodium vanadate for plasma membrane ATPase) were not performed. Consequently, the conclusion that PFOS specifically targets chloroplast ATP synthase is presented as a hypothesis supported by the collective evidence (differential Mg^2+^- vs. Ca^2+^-dependent activity, reduced intracellular ATP, and proteomic downregulation of ATP synthesis-related proteins), rather than as a definitive mechanistic finding.

### 3.4. PFOS Triggers Compensatory Photosynthetic Gene Expression but Suppresses ATP Synthesis and Redox Balance

The isolated chloroplast assays demonstrated that PFOS inhibits Mg^2+^-dependent ATP hydrolytic activity, reduces photophosphorylation efficiency, and interferes with thylakoid membrane electron transport. To further elucidate the molecular underpinnings, transcriptomic and proteomic analyses were performed on *E. gracilis* exposed to 50.0 μg/L PFOS for 7 days.

**Figure 8:**
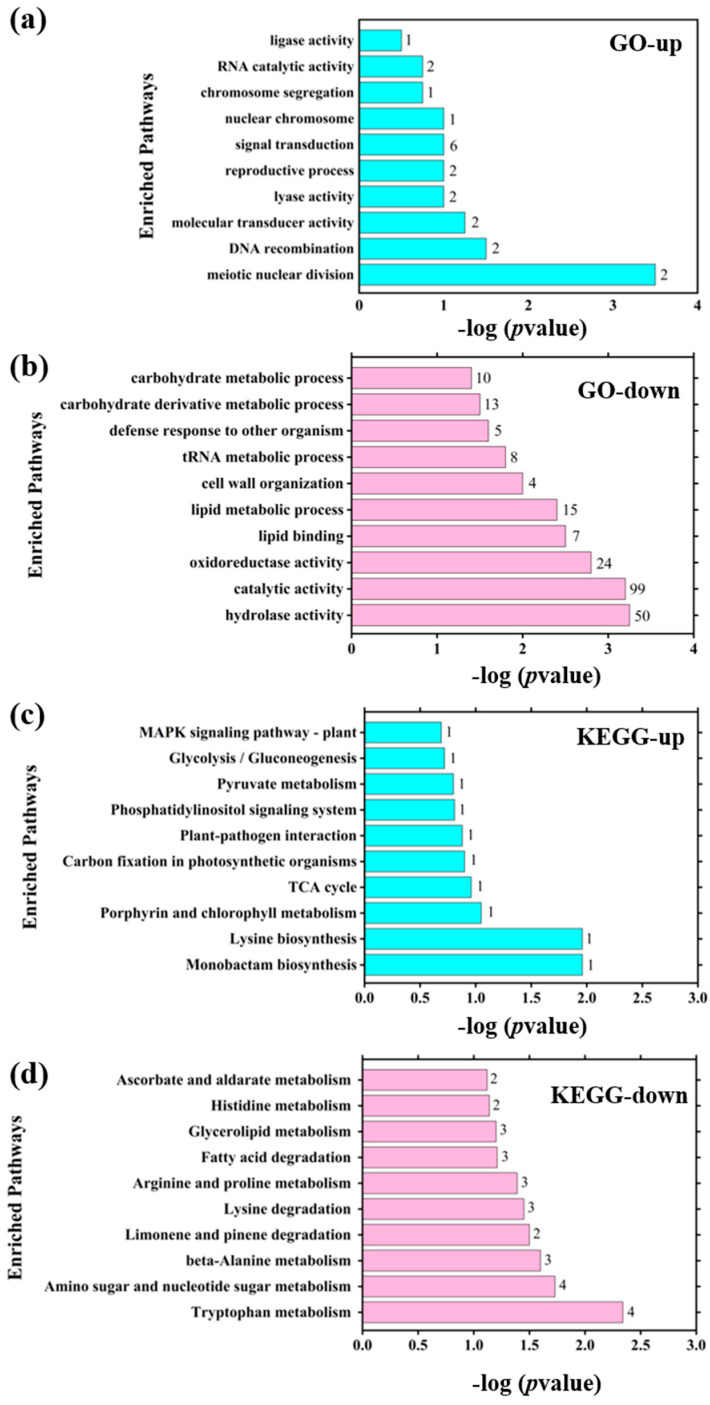
GO and KEGG enrichment analyses of differentially expressed genes in *E. gracilis* exposed to 50.0 μg/L PFOS for 7 days. (**a**) GO enrichment of upregulated genes. (**b**) GO enrichment of downregulated genes. (**c**) KEGG enrichment of upregulated genes. (**d**) KEGG enrichment of downregulated genes. The numbers in the bars correspond to the number of genes enriched in each pathway (for (**a**,**c**): number of upregulated genes; for (**b**,**d**): number of downregulated genes). Only significantly enriched terms (*p* < 0.05) are shown.

Transcriptome analysis identified 252 significantly differentially expressed genes (|log_2_FC| > 2, *p* < 0.05), of which 230 were downregulated and only 22 were upregulated, indicating that prolonged PFOS exposure leads to a predominantly suppressive transcriptional regulation. GO enrichment analysis (Figure 8a,b) showed that the differentially expressed genes were mainly involved in oxidoreductase activity, lipid metabolism, carbohydrate metabolism, and catalytic activity. KEGG enrichment analysis (Figure 8c,d) further revealed that PFOS affects amino acid metabolism, lipid metabolism, photosynthesis, chlorophyll metabolism, the TCA cycle, and the phosphatidylinositol signaling pathway. Notably, the upregulation of genes related to photosynthesis and chlorophyll metabolism is consistent with the increased chlorophyll content (Figure S6) and elevated *Fv*/*Fm* (Figure 5a) observed physiologically, suggesting that cells initiate a compensatory response at the transcriptional level to enhance light capture, rather than indicating a genuinely efficient photosynthetic state.

**Figure 9:**
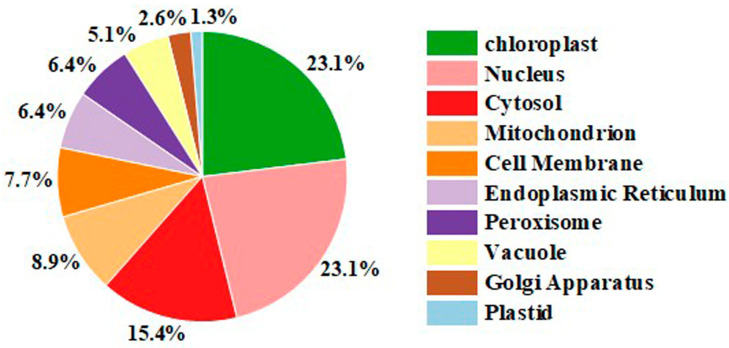
Subcellular localization analysis of differentially expressed proteins in *E. gracilis* exposed to 50.0 μg/L PFOS for 7 days. The numbers indicate the count of proteins localized to each compartment.

**Figure 10:**
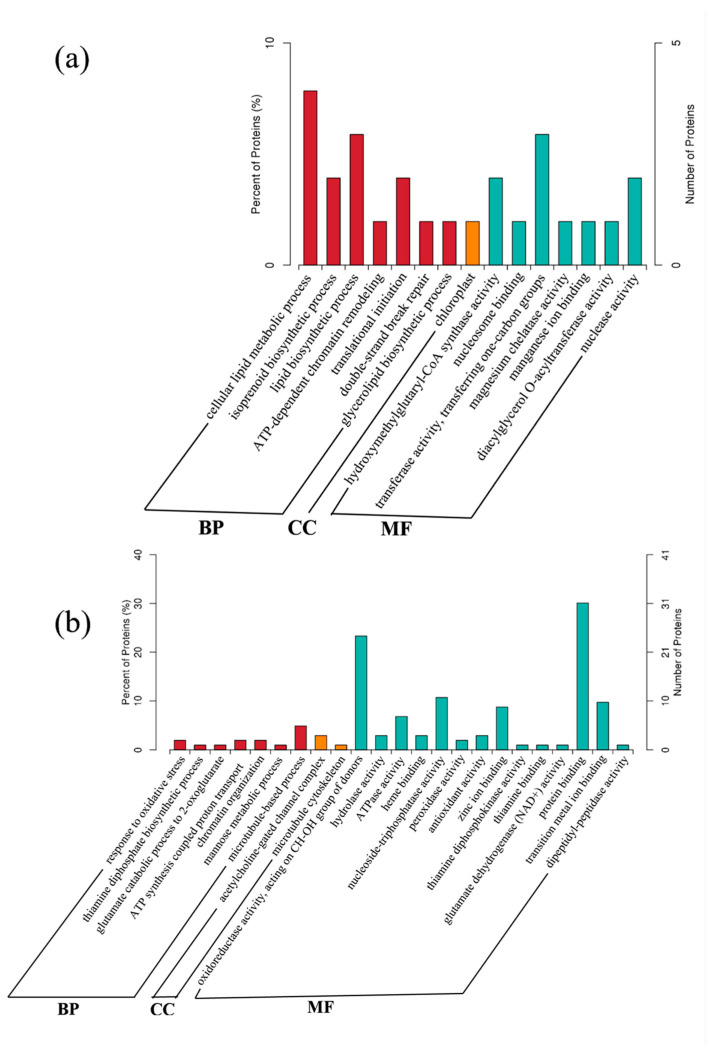
GO functional enrichment analysis of differentially expressed proteins in *E. gracilis* exposed to 50.0 μg/L PFOS for 7 days, categorized into biological process (BP), cellular component (CC), and molecular function (MF) terms. (**a**) Enrichment of upregulated proteins. (**b**) Enrichment of downregulated proteins. The numbers in the bars correspond to the number of proteins enriched in each GO term. Only significantly enriched terms (*p* < 0.05) are shown.

The proteomic data provided more direct molecular evidence. Subcellular localization analysis of differentially expressed proteins (fold change ≥ 1.2, *p* < 0.05, Benjamini–Hochberg corrected) (Figure 9) showed that the chloroplast was the most significantly affected compartment, in excellent agreement with the isolated chloroplast assays. GO enrichment analysis (Figure 10) revealed that upregulated proteins were mainly enriched in chloroplast function, lipid metabolism, and magnesium ion binding, whereas downregulated proteins were significantly enriched in ATP synthesis, peroxidase activity, and zinc/transition metal ion binding. Among these, the downregulation of ATP synthesis-related proteins directly corroborates the decreased Mg^2+^-ATP synthase activity observed in isolated chloroplasts (Figure 7d) and is consistent with the reduced intracellular ATP levels (Figure 6). This reveals a molecular imbalance characterized by “upstream enhancement but downstream blockage”: although genes involved in light capture and chlorophyll metabolism are upregulated, the terminal step of energy conversion—ATP synthase function—is impaired, leading to a decoupling between light capture and energy utilization. Previous studies have shown that when ATP synthase function is compromised, photosynthetic organisms upregulate the expression of light-harvesting complexes via feedback mechanisms [48]; however, such compensation cannot restore the loss of energy conversion efficiency. KEGG pathway enrichment analysis of the proteomic data (Figure S7) further revealed that proteins involved in photosynthetic processes were increased, whereas those related to carotenoid biosynthesis were decreased. The upregulation of photosynthetic proteins is consistent with the increased chlorophyll content (Figure S6) and elevated *Fv*/*Fm* (Figure 5a), reinforcing the view that cells mount a compensatory response to enhance light capture. In contrast, the downregulation of carotenoid biosynthesis may affect eyespot function (as the eyespot is rich in carotenoids) and photoprotection, although *NPQ* was elevated (Figure 5b).

The changes in metal ion-binding proteins further explain the observed increase in membrane interface resistance. Upregulation of magnesium-binding proteins reflects the cellular demand for chlorophyll synthesis and stabilization of photosynthetic structures, whereas downregulation of zinc- and transition metal-binding proteins suggests impaired antioxidant defense and partial electron transport dysfunction. This pattern is consistent with the concentration-dependent biphasic response of reactive oxygen species (ROS) levels (Figure 5d): at low PFOS concentrations, a slight decrease in ROS may be attributed to moderate activation of the antioxidant system; at high concentrations, the reduction in transition metal-binding proteins weakens oxidative stress defense, leading to ROS accumulation and subsequent membrane lipid peroxidation. Lipid peroxidation not only alters thylakoid membrane fluidity and permeability but may also increase membrane “stiffness” through oxidative cross-linking, thereby directly raising the resistance to ion transmembrane migration, as reflected by the increased membrane interface resistance. The agreement between omics expression patterns and physiological measurements (chlorophyll fluorescence parameters, photophosphorylation activity, intracellular ATP levels) supports the regulatory pattern of upstream photosynthetic pathway upregulation with downstream energy transduction impairment.

Beyond the photosynthetic system, multi-omics results also showed that PFOS significantly affects global metabolic networks. The TCA cycle, various amino acid metabolism pathways, and nitrogen metabolism were generally suppressed, indicating that the impact of PFOS extends from chloroplasts to mitochondria and overall cellular metabolism. Enrichment of pathways related to vesicle-mediated transport, lipid degradation, and ABC transporter proteins suggests that cells initiate damage repair and toxin efflux mechanisms. These findings are consistent with the increased flagellar shedding rate, reduced apparent migration velocity, and altered electrochemical characteristics of thylakoid membranes, indicating that PFOS acts at multiple levels, affecting both membrane systems and energy metabolism.

Taken together, the results form a self-consistent explanatory framework. PFOS accumulates in the Euglena eyespot, causing phototactic abnormalities and impaired motility—a direct disturbance of the photoreceptive-motor system. In vivo PSII parameters did not decline with eyespot damage; instead, chlorophyll content, *Fv*/*Fm*, and *NPQ* increased, indicating that changes in the photosynthetic system cannot be attributed to eyespot impairment. Isolated chloroplast assays demonstrated that PFOS inhibits Mg^2+^-dependent ATP hydrolytic activity and impairs electron transport efficiency and Mg^2+^-ATP synthase activity at the chloroplast level. Transcriptomic and proteomic results further revealed that *E. gracilis* initiates a compensatory response characterized by upregulation of photosynthesis-related processes under PFOS stress, but the downregulation of ATP synthesis-related proteins prevents recovery of energy conversion efficiency, resulting in a decoupled state of “upstream enhancement and downstream blockage”.

## 4. Conclusions

This study systematically investigated the effects of environmentally relevant PFOS at concentrations spanning from environmentally typical (0.5 µg/L) to supra-environmental (50 µg/L) on phototaxis, photosynthetic function, and energy metabolism in the photoautotrophic flagellate *E. gracilis*. The main findings are as follows:

(i) TEM-EDS mapping revealed pronounced fluorine signal enrichment (attributable to PFOS) in the eyespot, chloroplasts, and mitochondria of PFOS-exposed cells. This enrichment was associated with altered phototactic responses, increased flagellar shedding, and reduced apparent migration velocity, indicating disturbance of the photoreceptive and motor systems. It should be noted that the TEM-EDS mapping is qualitative and indicates relative enrichment patterns rather than quantitative subcellular PFOS concentrations.
(ii) At environmentally typical to hotspot-relevant concentrations (0.5–5 µg/L), PFOS did not acutely inhibit PSII photochemical efficiency; instead, chlorophyll content, *Fv*/*Fm*, and *NPQ* were elevated, suggesting activation of a compensatory photoprotective response at the PSII level. However, intracellular ATP levels progressively declined, while ROS showed a concentration-dependent biphasic pattern. Growth displayed weak promotion at low concentrations and an inhibitory trend only at the highest concentration (50.0 µg/L) by day 10. The divergence between enhanced PSII parameters and reduced ATP output indicates that the PFOS-induced upregulation represents stress-related compensation rather than genuine functional improvement.
(iii) Isolated chloroplast assays demonstrated that PFOS inhibits Mg^2+^-dependent ATP hydrolytic activity in the chloroplast-enriched fraction and impairs electron transport efficiency, accompanied by altered thylakoid membrane electrochemical characteristics. Integrative transcriptomic and proteomic analyses revealed a molecular pattern of upregulated photosynthesis- and chlorophyll metabolism-related genes/proteins alongside downregulated ATP synthesis-related proteins, reflecting a compensatory response of “upstream enhancement but downstream blockage.” The differential sensitivity of Mg^2+^-dependent versus Ca^2+^-dependent activities is consistent with impaired chloroplast ATP synthase function, though the specific molecular target and mechanism remain to be conclusively demonstrated.

Collectively, PFOS disrupts light resource utilization in *E. gracilis* through dual interference at the behavioral and physiological levels. At the behavioral level, fluorine signals attributed to PFOS were prominently detected in the eyespot, and this enrichment was associated with impaired phototaxis and motility, suggesting a compromised ability of cells to actively locate optimal light environments. At the physiological level, PFOS inhibits Mg^2+^-dependent ATP hydrolytic activity in the chloroplast-enriched fraction and suppresses photophosphorylation, and reduces ATP output. These findings are consistent with impaired chloroplast ATP synthase function; however, the specific molecular target and the precise mechanism of impairment remain to be conclusively identified. Cells mount a compensatory upregulation of photosynthetic processes in response, but this cannot overcome the bottleneck imposed by impaired energy transduction, ultimately leading to a decoupled state and reduced effective utilization of light resources. The proposed mechanistic model linking chloroplast ATP synthase functional impairment to reduced ETR remains a hypothesis warranting direct experimental validation using uncoupler treatments and phosphorylating versus non-phosphorylating conditions. These findings contribute to the risk assessment of PFAS in aquatic ecosystems and highlight the value of Euglena as a model for evaluating multi-level toxic effects.

## Data Availability

The original contributions presented in this study are included in the article/Supplementary Material. Further inquiries can be directed to the corresponding author.

## Supplementary Materials

The following supporting information can be downloaded at https://www.mdpi.com/article/10.3390/toxics14060540/s1, Figure S1: (a) FT-IR spectra of PFOS and PTFE; (b) AFM IR spectra of Euglena before and after PFOS exposure: (E0) unexposed control; (E3) exposed to 50.0 μg/L PFOS; Figure S2: Estimated PPFD gradient along the migration channel; Figure S3: Optical (a) and fluorescence (b) microscopy images of isolated chloroplasts; Figure S4: The equivalent circuit model used for fitting EIS data; Figure S5: Microscopic observation of flagellar shedding in PFOS treated cells; Figure S6: Chlorophyll content in *E. gracilis* exposed to different concentrations of PFOS (0, 0.5, and 50.0 μg/L); Figure S7: KEGG enrichment bubble plot of differentially expressed proteins.
