# Supplementary material for: Environmental Concentrations of PFOS Accumulate in the Euglena Eyespot and Impair Chloroplast ATP Synthase Activity: A Dual Impairment of Phototaxis and Photosynthetic Light Reactions
Source: Toxics. 2026 Jun 22;14(6):540. doi: 10.3390/toxics14060540 (PMC13308086; doi:10.3390/toxics14060540)
Supplement: Supplementary file 1 [file toxics-14-00540-s001.zip › toxics-4298030-supplementary.pdf]

Supporting Information for

Environmental concentrations of PFOS accumulate in the *Euglena* eyespot and impair chloroplast ATP synthase activity: A dual impairment of phototaxis and photosynthetic light reactions

Peirui Liu[1*], Junfeng Wang[1], Yan Hong[1], Zilin Chen[1], Xiaoya Liu[2], Huayi Chen[3], Ganning Zeng[1], Xiangliang Pan[1]

[1] Zhejiang Key Laboratory of Low-carbon Control Technology for Industrial Pollution, College of Environment, Zhejiang University of Technology, Hangzhou 310014, China

[2] School of Environmental Science and Engineering, Xiamen University of Technology, Xiamen 361024, China

[3] School of Tropical Agriculture and Forestry, Hainan University, Haikou, 570228, China

[*]Corresponding Author, Email address: liupeirui1013@zjut.edu.cn

The Supporting Information includes seven figures.

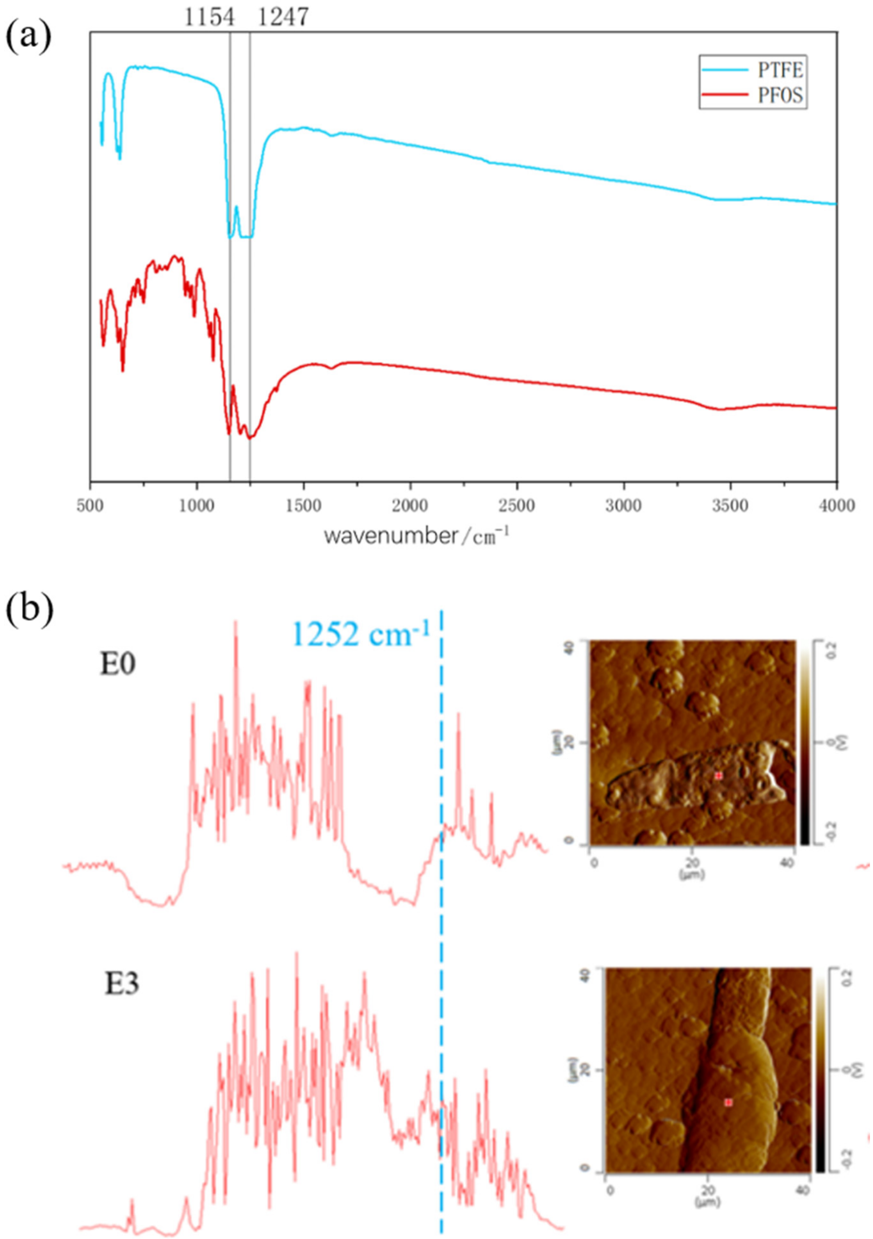

Figure S1. (a) FT-IR spectra of PFOS and PTFE; (b) AFM-IR spectra of *Euglena* before and after PFOS exposure: (E0) unexposed control; (E3) exposed to 50.0 μg/L PFOS. The peak at 1252 cm^{-1} corresponds to the C–F bond.

Figure S1a shows the Fourier transform infrared (FT-IR) spectra of perfluorooctane sulfonate (PFOS) and polytetrafluoroethylene (PTFE). PTFE only contains C–C and C–F bonds, while PFOS possesses other functional groups such as

thiol groups. To eliminate the interference from these functional groups, we compared the FT-IR profiles of PFOS and PTFE, and confirmed that the characteristic absorption peak of C–F bonds in PFOS appears at approximately 1247 cm^{-1}. To verify whether the infrared absorption peaks shift after PFOS binds to the cell membrane, we performed point scanning on the surface of *Euglena gracilis* cells using AFM-IR before and after PFOS exposure. The results are presented in Figure S1b. The acyl absorption peak of algal phospholipids shifted from 1732 cm^{-1} to 1728 cm^{-1}. The amide II band was detected at 1644 cm^{-1}, and the absorption peak of C–F bonds was located at 1252 cm^{-1}.

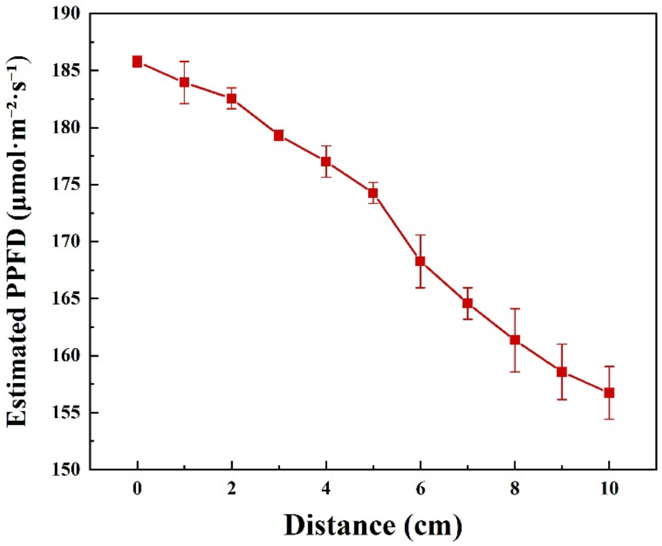

Figure S2. Estimated PPFD gradient along the migration channel. Irradiance ($W \cdot m^{-2}$) was measured using a solar power meter (SM206-SOLAR) at 1-cm intervals and converted to PPFD using an empirical conversion factor for the 6500 K cool-white LED. The monotonic decrease confirms a continuous light gradient from the light-proximal to the light-distal end.

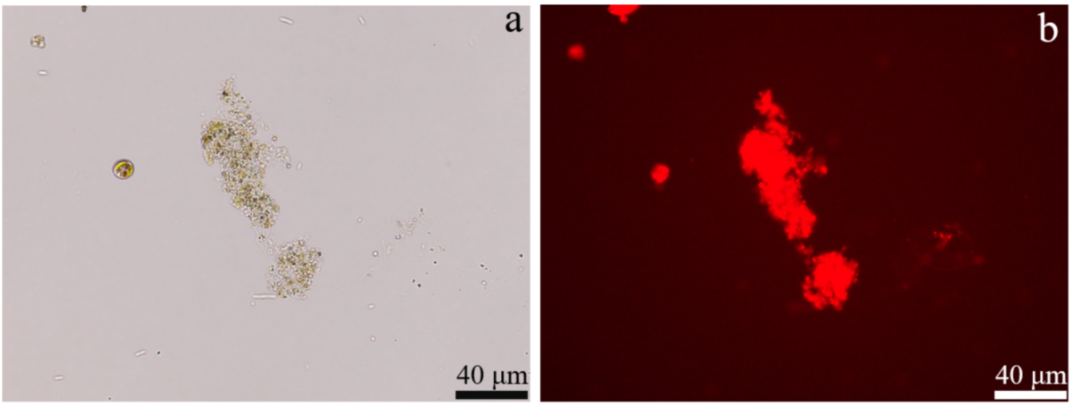

Figure S3. Optical (a) and fluorescence (b) microscopy images of isolated chloroplasts.

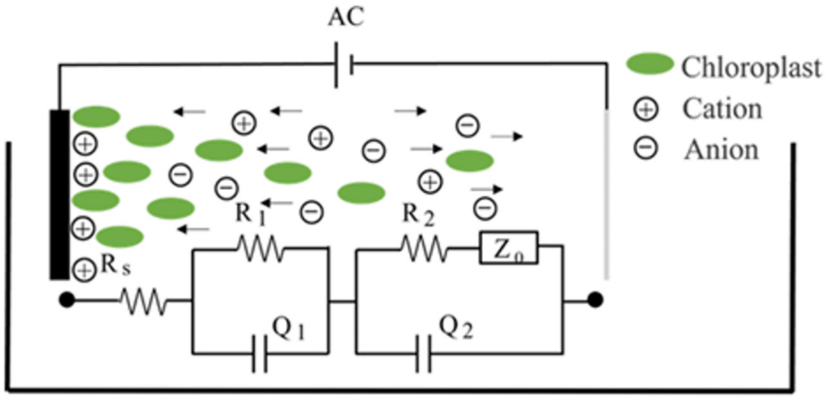

Figure S4. The equivalent circuit model used for fitting EIS data. The circuit comprises a solution resistance (R_s) in series with a membrane/interface resistance (R_1), a charge transfer resistance (R_2), and a Warburg impedance (Z_0) connected in series with R_2. In this model, R_s represents the uncompensated ohmic resistance of the electrolyte and electrode leads.

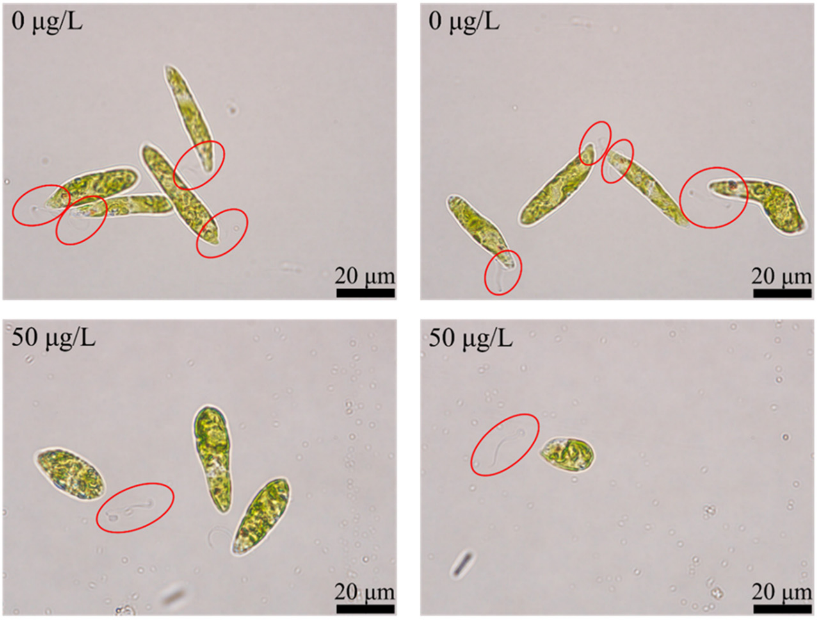

Figure S5. Microscopic observation of flagellar shedding in PFOS-treated cells.

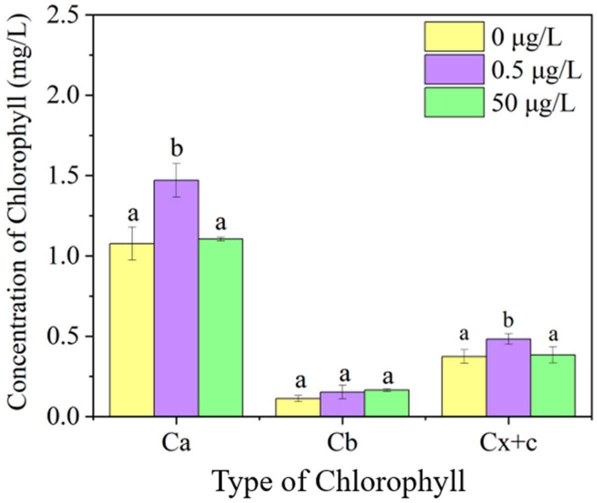

Figure S6. Chlorophyll content in *E. gracilis* exposed to different concentrations of PFOS (0, 0.5, and 50.0 μg/L). Data are presented as mean ± SD (n = 3). Different lowercase letters indicate significant differences among groups ($p < 0.05$).

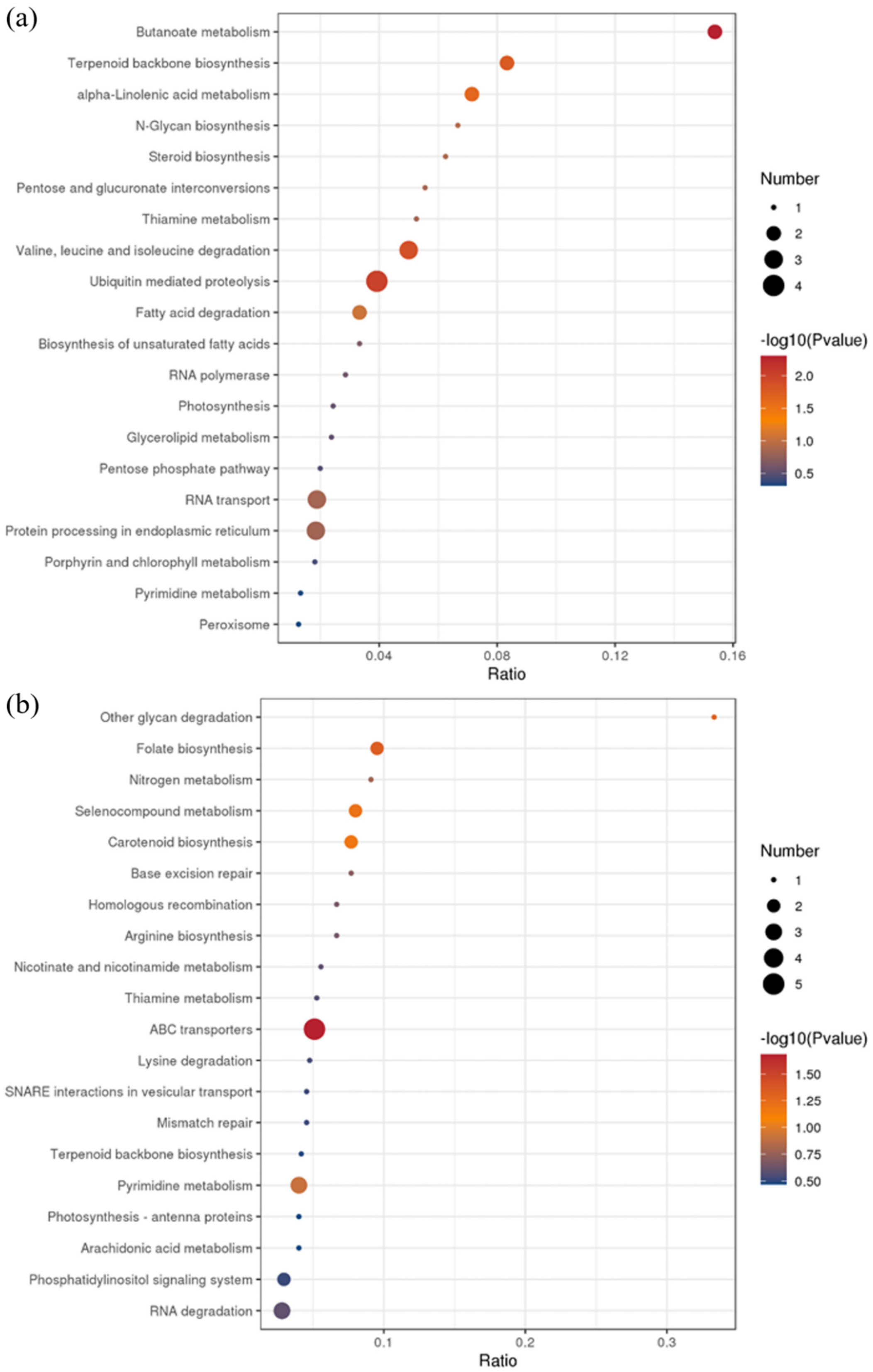

Figure S7. KEGG enrichment bubble plot of differentially expressed proteins. (a) Upregulated. (b) Downregulated. Bubble size: number of proteins per pathway.
